# A Fentanyl‐Responsive Microneedle Patch for Harm Reduction

**DOI:** 10.1002/advs.202524301

**Published:** 2026-07-09

**Authors:** Penghui Zhao, Zerui Zhou, Tyler Wolter, Huanqing Niu, Yuanzhi Bian, Chixia Tian, Chun Xu, Chenming Zhang, Juhong Chen, Matthew W. Buczynski, Wujin Sun

**Affiliations:** ^1^ Department of Biological Systems Engineering Virginia Tech Blacksburg Virginia USA; ^2^ Academy of Integrated Science, College of Science Virginia Tech Blacksburg Virginia USA; ^3^ School of Pharmacy University of Wisconsin‐Madison Madison Wisconsin USA; ^4^ Sydney Dental School The University of Sydney Camperdown New South Wales Australia; ^5^ Department of Bioengineering University of California Riverside Riverside California USA; ^6^ School of Neuroscience Virginia Tech Blacksburg Virginia USA

**Keywords:** Fentanyl, Harm Reduction, Microneedle, Naloxone, Responsive Delivery

## Abstract

The efficacy of current opioid overdose interventions is fundamentally limited by their reliance on bystander detection and administration, leaving unwitnessed overdoses, a predominant cause of fatalities, unaddressed. Herein, we developed an innovative fentanyl‐responsive microneedle (MN) patch (iNal patch) engineered as an autonomous harm reduction tool to dynamically release naloxone on demand in response to fentanyl levels. Specifically, the iNal patch integrates mesoporous silica nanoparticles (MSNs) loaded with naloxone. The nanoparticle surfaces are modified by fentanyl‐sensitive aptamers, allowing precise and dose‐dependent drug release triggered by fentanyl exposure. The engineered MN matrix composed of swellable maleated poly(vinyl alcohol) facilitates rapid skin penetration and interstitial fluid access, ensuring immediate and sustained naloxone release. From in vitro and in vivo studies, the iNal patch was demonstrated to effectively reverse fentanyl‐induced opioid overdose symptoms, rapidly restore normal physiological behaviors in mice, and enable multiple responsive drug‐release cycles to prevent renarcotization. This proof‐of‐concept MN platform establishes a new paradigm for materials‐based harm reduction, offering an autonomous safety net for high‐risk populations independent of human supervision.

## Graphical abstract

A fentanyl‐responsive microneedle patch is developed as a self‐regulating overdose intervention that releases naloxone upon detecting fentanyl. The system employs aptamer‐functionalized nanoparticles embedded in a swellable polymer matrix to enable rapid, on‐demand drug delivery through the skin. This platform provides a continuous, autonomous safeguard against overdose without requiring external monitoring or intervention.

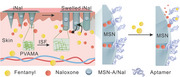

## 1 Introduction

The opioid overdose crisis is a severe global health issue, causing over 107,000 deaths in the U.S. in 2022 [1, 2, 3]. Synthetic opioids such as fentanyl are particularly devastating, accounting for 90% of these fatalities [4]. Opioid overdose results in profound respiratory depression through µ‐opioid receptor activation, which can cause loss of consciousness and potentially death [5]. Naloxone, a potent opioid antagonist, is widely recognized as the most effective emergency antidote [6, 7, 8]. However, current harm reduction strategies, including the widespread distribution of intranasal naloxone, suffer from a critical limitation: they fundamentally rely on the presence of a bystander to detect the overdose and administer the antidote [9, 10, 11]. This dependency leaves individuals using opioids alone [12], a common scenario in fatal overdoses, completely unprotected. Furthermore, therapeutic efficacy is constrained by the short duration of action compared to fentanyl, the necessity of multiple timely administrations [13, 14, 15], and the risks associated with non‐medical or delayed administration.

To bridge the gap between unwitnessed overdose events and life‐saving intervention, recent innovations aim to transform harm reduction from a reactive, bystander‐dependent model to an autonomous, prophylactic system. Implantable devices, such as the iSOS device and Naloximeter [16, 17], continuously monitor physiological parameters like respiratory rate, blood oxygen saturation, and heart rate, and autonomously trigger naloxone release upon overdose detection. Similarly, wearable injector systems employing accelerometer‐based sensors or electrocardiography monitors detect overdose‐related apnea or respiration decline, and automatically administer naloxone subcutaneously [18, 19]. While these systems improve autonomy, they rely on indirect physiological markers, which can limit the timeliness and precision of intervention [16]. Physiological markers reflect opioid exposure indirectly and often with delayed sensitivity, potentially compromising optimal therapeutic outcomes [20]. To function as a true prophylactic safety net, a device must transcend reactive physiological monitoring. It requires a direct, chemical‐responsive mechanism capable of preventing overdose progression before respiratory depression becomes irreversible. Such a platform would effectively serve as an “autonomous bystander”, providing on‐demand protection to high‐risk populations without requiring abstinence or active user compliance.

**FIGURE 1:**
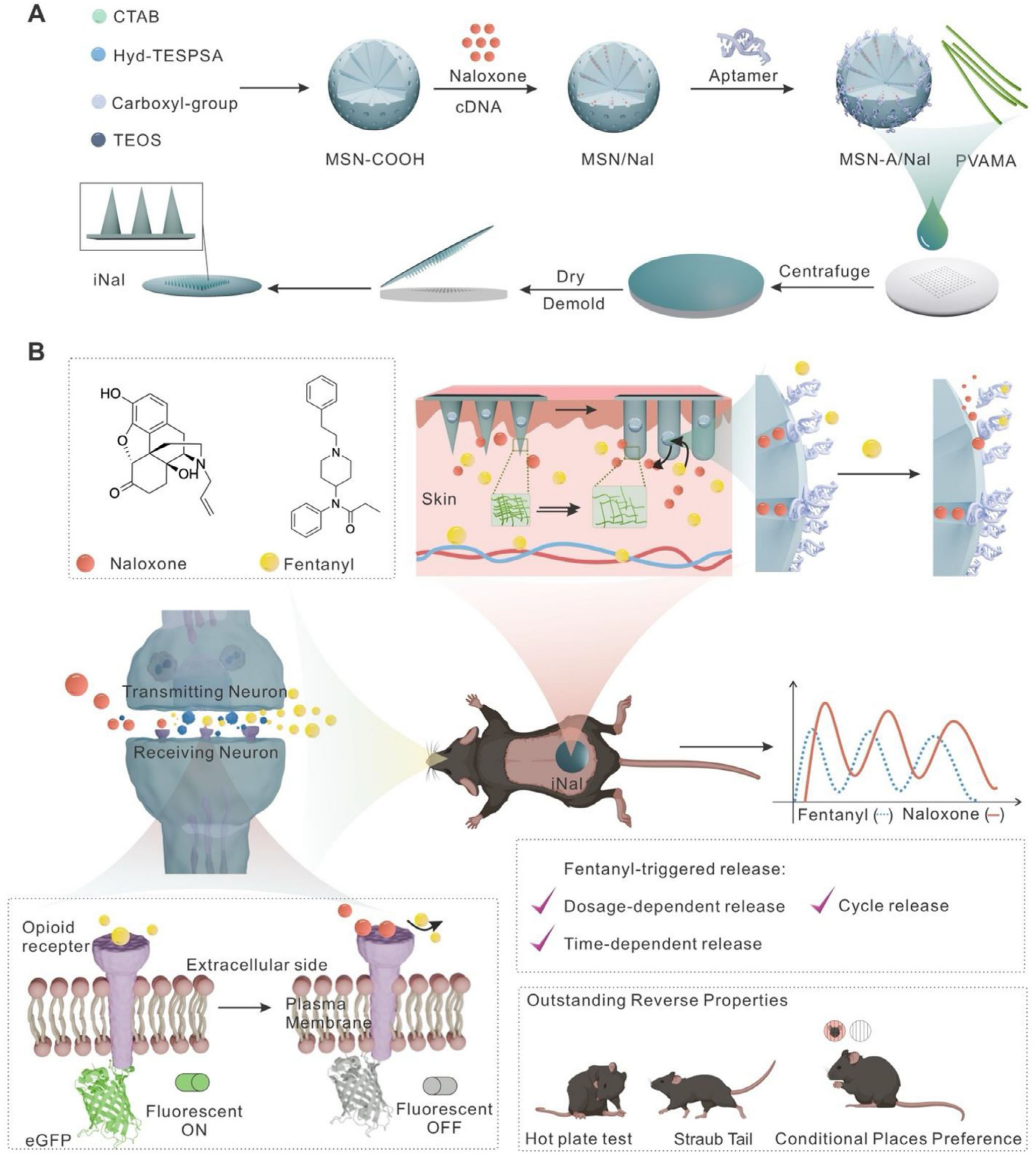
Design and chemo‐mechanical actuation of the fentanyl‐responsive iNal patch. (A) Fabrication of the iNal patch via micromolding, where naloxone‐loaded mesoporous silica nanoparticles (MSN‐A/Nal) are embedded within a swellable maleated poly(vinyl alcohol) (PVAMA) microneedle matrix. (B) Schematic of the mechanism of action in vivo. Upon skin insertion, the hydrogel matrix serves as a dynamic conduit, rapidly swelling to facilitate the influx of interstitial fluid and fentanyl molecules to the distributed nanovalve network. The MSNs are capped with fentanyl‐specific aptamers that function as responsive molecular gates. The competitive binding of physiological fentanyl to these aptamers triggers the specific "unlocking" of the nanopores, resulting in the proportional and sustained release of the antidote (naloxone). This autonomous response effectively reverses opioid‐induced symptoms and enables multi‐cycle release to prevent renarcotization.

Developing such a materials‐based harm reduction tool requires overcoming several challenges: (1) rapid in vivo fentanyl‐dependent response; (2) sufficient naloxone supply to reverse lethal doses; (3) small, portable design that is simple to inject; (4) feasibility for mass‐manufacturing; and (5) high biocompatibility with no short‐term or long‐term toxicity. In this study, we present a fentanyl‐responsive microneedle patch (iNal) for autonomous overdose reversal (Figure 1). The iNal patch integrates mesoporous silica nanoparticles (MSNs) loaded with the opioid antagonist naloxone and capped by aptamers previously reported to exhibit high specificity and affinity for fentanyl [21, 22, 23]. These aptamers serve as responsive molecular gates, enabling direct sensing of fentanyl in physiological conditions. Fentanyl binding opens the gate and proportionally releases naloxone. This nanoparticle design uniquely permits repeated and controlled drug release cycles, providing a sustained defense against renarcotization [24], a phenomenon where fentanyl outlasts the antidote, thereby overcoming limitations associated with single‐dose delivery systems.

To facilitate convenient administration, the functionalized nanoparticles are incorporated into an MN array composed of maleated poly(vinyl alcohol) (PVAMA), a biocompatible, swellable polymer. Upon skin application, the PVAMA MNs rapidly swell, enhancing interstitial fluid uptake and enabling rapid diffusion and sensitive detection of fentanyl. The iNal patch demonstrates robust mechanical strength, effective skin penetration, and excellent biocompatibility, ensuring safe and efficient transdermal drug administration. In vitro and in vivo evaluations confirmed the rapid and repeated fentanyl‐triggered release of naloxone, effectively reversing opioid‐induced physiological and behavioral symptoms. By bridging the gap between unwitnessed overdose events and life‐saving intervention, the iNal patch establishes a new paradigm in harm reduction: the “autonomous bystander”. Unlike reactive systems that depend on external detection, or electronic monitors limited by physiological lag times, this materials‐based platform enables prophylactic protection. It functions as a chemical sentinel, detecting systemic fentanyl and initiating counter‐measures aimed at arresting the overdose trajectory prior to the onset of severe physiological collapse, thereby offering a robust safety net for high‐risk populations independent of human supervision or active compliance.

## 2 Results

### 2.1 Preparation and Characterization of Aptamer‐Gated Naloxone‐Loaded MSNs

**FIGURE 2:**
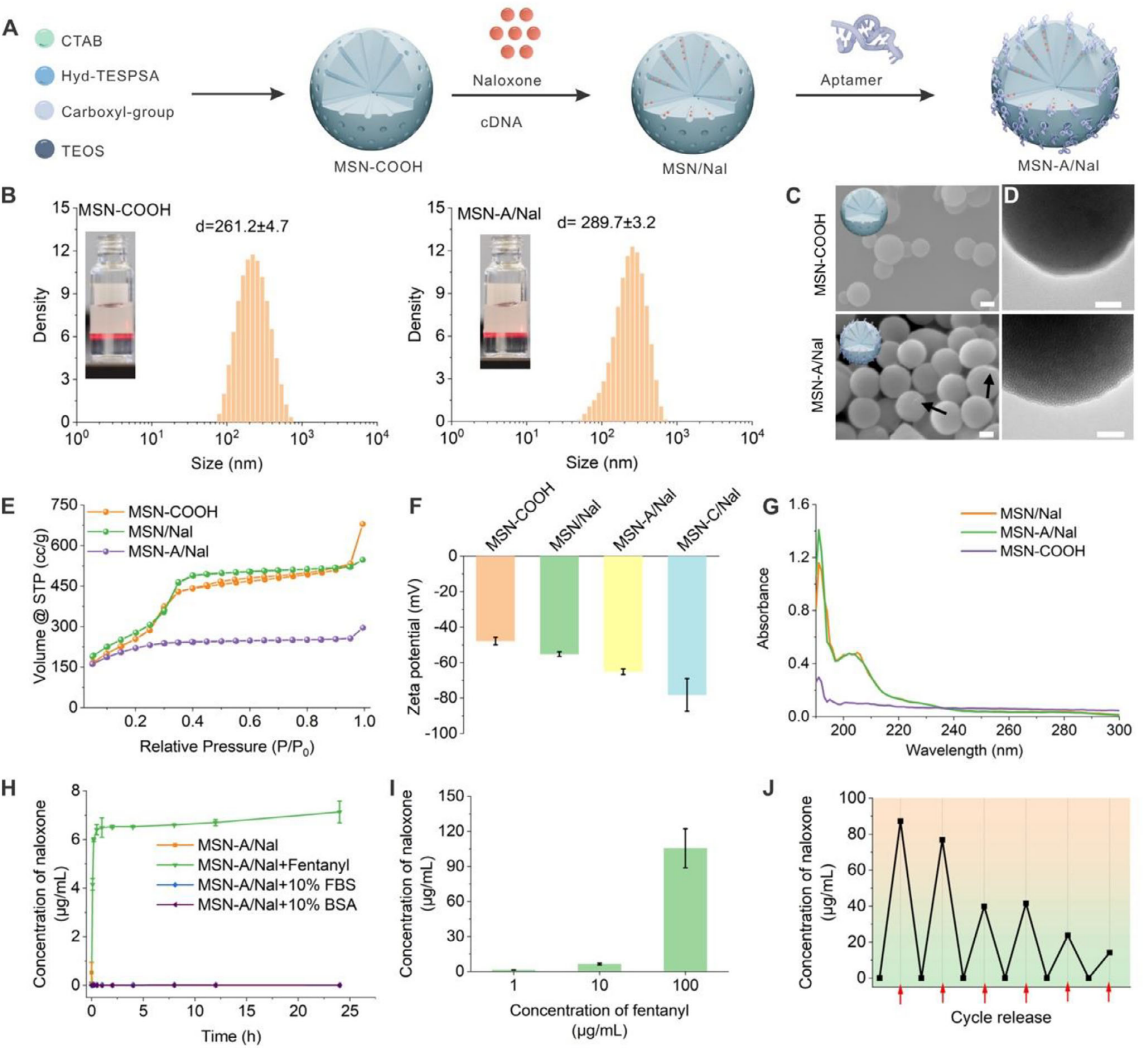
Fabrication and characterization of MSNs. (A) Schematic diagram of the synthetic MSN‐A/Nal procedure. First surfactant‐assembly sol‐gel process was used to prepare carboxyl‐functionalized mesoporous silica nanoparticles (MSN‐COOH), which was used as a material template for cDNA conjugation. Naloxone was then loaded into the channels of MSN, and the openings of the channels were capped by aptamers binding to cDNAs. (B) Particle size distribution of MSNs via DLS. The insets show the aqueous solutions of MSNs. (C) SEM of MSNs, Scale bar. 200 nm. (D) TEM images of MSNs., Scale bar, 50 nm. (E) Nitrogen adsorption/desorption isotherms curves of MSNs. (F) Zeta potential data showing the degree of dispersion and surface electrical properties of the MSNs. (G) UV–Vis spectrum of MSNs. (H–J) In vitro accumulated naloxone release from MSNs (H) at different time points, (I) under different fentanyl concentrations, and (J) cycle release of naloxone when triggered by fentanyl from MSN‐A/Nal. Data are presented as mean ± s.d. (n = 3).

To create a fentanyl‐responsive naloxone delivery platform, we first synthesized carboxyl‐functionalized mesoporous silica nanoparticles (MSN‐COOH) designed for efficient drug loading and surface modification. To impart fentanyl sensitivity, MSNs were functionalized with a complementary DNA (cDNA) strand, loaded with naloxone (forming MSN/Nal), and subsequently capped by hybridizing a fentanyl‐specific aptamer to the cDNA (forming MSN‐A/Nal), creating a gatekeeper for the nanopores (Figure 2A). Dynamic light scattering (DLS) tracked particle size change. The hydrodynamic diameter increased from 261.2 ± 4.7 nm for the initial MSN‐COOH to 289.7 ± 3.2 nm for the final MSN‐A/Nal construct, with an intermediate size of 286.5 ± 5.8 nm observed for MSN/Nal (Figure 2B, Figure S1A, and Table S1). This ∼25–30 nm increase confirms successful surface modification with cDNA/aptamer and the loading of naloxone. Morphological analysis by scanning electron microscopy (SEM) revealed spherical nanoparticles with a distinct multichannel structure indicative of their mesoporous nature (Figure 2C and Figure S1B), while transmission electron microscopy (TEM) further showed the successful functionalization of the MSN surface (Figure 2D and Figure S1C).

Next, we characterized the mesoporous structure critical for drug loading and release using N_2_ adsorption–desorption analysis [25]. The bare MSN‐COOH exhibited a high specific surface area (S_BET_ = 1066 m^2^·g^−1^) and substantial pore volume (V_P_ = 0.44 cm^3^·g^−1^) with a pore diameter (D_P_) of 3.8 nm, typical for mesoporous materials (Figure 2E, Figure S2, and Table S1). Following naloxone loading (intermediate step), the surface area and pore volume decreased significantly to 545 and 0.12 cm^3^·g^−1^, respectively, while the pore diameter remained similar (3.4 nm), strongly suggesting successful naloxone encapsulation within the MSN channels. Subsequent aptamer capping (MSN‐A/Nal) resulted in a further slight decrease in surface area (459 m^2^·g^−1^) and pore volume (0.09 cm^3^·g^−1^) but a marked reduction in pore diameter to 1.0 nm. This significant decrease in pore diameter strongly indicates that the hybridized aptamer effectively capped the pore entrances, crucial for preventing premature drug leakage. Our results are consistent with other studies that use MSN for drug delivery [26].

Surface chemistry modifications achieved under these coupling conditions were definitively confirmed using zeta potential measurements and Energy Dispersive Spectroscopy (EDS). The zeta potential progressively increased in negative charge from −47.8 ± 2.1 mV (MSN‐COOH) to −55.2 ± 1.3 mV (MSN/Nal) and finally to −65.1 ± 1.6 mV for MSN‐A/Nal, consistent with the addition of negatively charged DNA aptamers (Figure 2F). EDS mapping corroborated aptamer conjugation by detecting nitrogen (N), and phosphorus (P) signals on the MSN‐A/Nal surface, elements absent in the initial MSN‐COOH which primarily showed oxygen (O) and silicon (Si) (Figure S3). UV–Vis spectroscopy further confirmed the aptamer conjugation (Figure 2G).

To evaluate drug loading and the specificity of the gating mechanism, we optimized the loading ratio (cDNA:naloxone:MSN = 0.1:1:1, Figure S4) and synthesized a control nanoparticle (MSN‐C/Nal) using a random DNA sequence instead of the fentanyl aptamer. MSN‐A/Nal achieved a high drug loading efficiency (DLE) of 85.31 ± 0.97% and a drug loading capacity (DLC) of 42.65 ± 0.48% (Table S1), this corresponds to 0.71 ± 0.06 nmol of aptamer per mg of MSN. Interestingly, the control MSN‐C/Nal showed slightly higher DLE (95.43 ± 2.01%) and DLC (47.72 ± 1.01%), possibly due to non‐specific interactions or different packing of the random DNA. Similar drug loading capacity in MSN‐based drug delivery systems has also been observed by others [27, 28].

#### 2.1.1 In Vitro Naloxone Release From MSN‐A/Nal

To assess the functional performance of the engineered nanoparticles, we first evaluated the in vitro naloxone release profile from MSN‐A/Nal upon fentanyl triggering. MSN‐A/Nal showed rapid, fentanyl‐triggered naloxone release in PBS, with an initial burst in ∼5 min and a peak at 1 h (Figure 2H). Importantly, the release kinetics were comparable when tested in a solution containing fetal bovine serum (FBS) or bovine serum albumin (BSA). While these media mimic the complex protein environment of physiological fluids, we utilized supra‐physiological challenge concentrations of fentanyl (1–100 µg/mL) to rigorously assess the dynamic range and saturation limits of the aptamer gating mechanism (Figure 2H). Naloxone release was clearly dependent on the fentanyl concentration; exposure to 10 µg/mL fentanyl resulted in a cumulative release of 6.49 ± 0.83 µg/mL naloxone, while a higher fentanyl concentration of 100 µg/mL triggered a significantly larger release of 105.50 ± 16.69 µg/mL (Figure 2I). This confirms the desired concentration‐responsive release mechanism. Furthermore, the naloxone release process lasts for up to 24 h, indicating potential for sustained delivery. We also investigated the potential for multi‐dose release by repeatedly exposing the MSN‐A/Nal to fentanyl. The results showed that the nanoparticles could indeed release naloxone over multiple fentanyl exposure cycles, with an approximate ∼40% reduction by the third cycle (Figure 2J). This multi‐dose capability, likely facilitated by the channel‐like architecture of the MSNs allowing gradual drug efflux and the progressive opening of the remaining capped pores upon subsequent triggers. This ensures a sustained therapeutic reserve crucial for preventing renarcotization (where opioid duration exceeds that of the antidote) or addressing repeated exposures [29], making the platform suitable for autonomous, prophylactic harm reduction.

#### 2.1.2 Fabrication and Characterization of the iNal Patch

**FIGURE 3:**
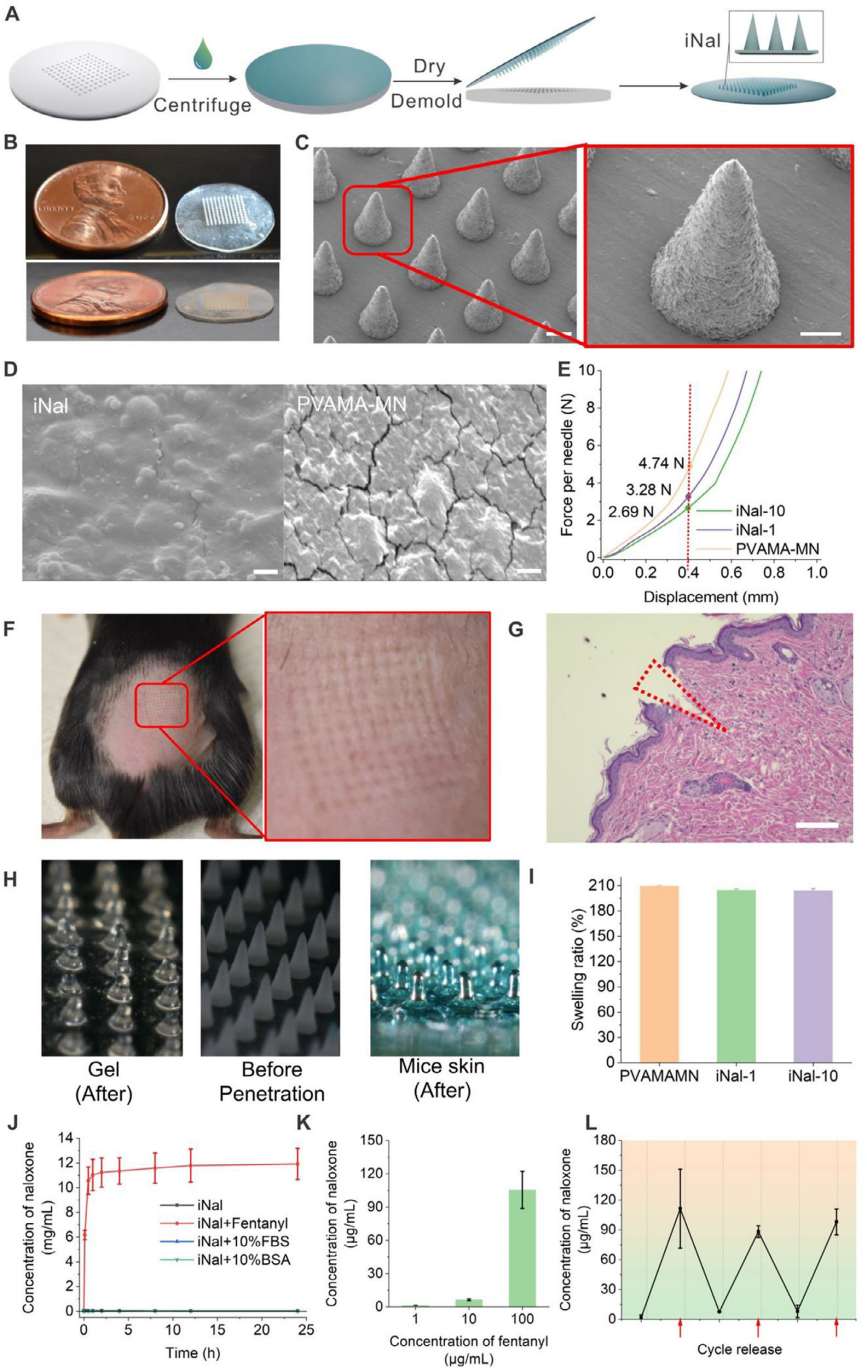
Fabrication and characterization of iNal. (A) Schematic illustration of iNal patch preparation. (B) Photography of the iNal patch compared with one cent coin. (C) SEM of iNal patch. Scale bar, 200 µm (Left), 100 µm (Right). (D) SEM of the surface from the iNal patch or PVAMA MN patches. Scale bar, 1 µm. (E) Mechanical properties of MNs. (F) Penetration of the iNal patch in mice skin. (G) H&E staining of skin. Scale bar, 200 µm. (H) Images of the iNal patch after swelling and recovery in water and mice skin. (I) The quantity of swelling properties of MNs. (J–L) In vitro accumulated naloxone release from MNs (J) at different time points, (K) under different fentanyl concentrations, and (L) cycle release of naloxone when triggered by fentanyl (100 µg/mL). Data are presented as mean ± s.d. (n = 3).

We embedded the MSNs in a swellable PVAMA MNs for transdermal delivery. MN arrays were fabricated using a micromolding technique (Figure 3A), resulting in patches containing an 11×11 array of conical needles, with the entire patch being compact (less than the size of a one‐cent coin) (Figure 3B). Each needle measured approximately 600 µm in height and 300 µm in diameter at the base, with an inter‐needle spacing of 300 µm. SEM images confirmed the successful fabrication of the MN array and showed a slightly more sealed surface morphology after loading the MSN‐A/Nal nanoparticles compared to plain PVAMA MNs, indicative of successful nanoparticle incorporation (Figure 3C,D).

Ensuring adequate mechanical strength for skin penetration is crucial for MN patch efficacy. We evaluated the compressive strength using an Instron tester (Figure 3E). The failure force required to buckle the needles was measured to be 4.74 N for plain PVAMA MNs, 3.28 N for iNal patch loaded with 1 mg naloxone/patch MSN‐A/Nal (iNal‐1), and 2.69 N for patches loaded with 10 mg naloxone/patch MSN‐A/Nal (iNal‐10). Nanoparticle loading slightly lowered strength, yet the failure forces remained within a range compatible with reliable skin insertion, as further corroborated by the effective penetration observed in subsequent histological analysis. The failure force value is consistent with our previous as well as others' reports [30, 31, 32]. Successful penetration into mouse skin *ex vivo* was visually confirmed (Figure 3F). Subsequent histological analysis using hematoxylin and eosin (H&E) staining revealed that the MNs achieved a penetration depth of approximately 400 µm, sufficient to reach the dermal layer for effective drug delivery (Figure 3G). Importantly, the skin tissue showed good recovery after patch removal (Figure S5), and no signs of significant irritation were observed over the following three days (Figure S6), indicating the biocompatibility and safety of the MN application.

Given the importance of matrix swelling in facilitating drug diffusion and release from hydrogel‐based MNs, we assessed the swelling properties of the PVAMA MNs [33, 34]. As the polymer matrix swells, its mesh size increases, which can accelerate the diffusion of both the trigger molecule (fentanyl) into the patch and the released drug (Nal) out of the patch. The MNs swelled markedly in water and skin (Figure 3H). Quantitative analysis revealed a substantial swelling ratio of approximately 200% (Figure 3I). This high degree of swelling is expected to enhance the release kinetics of naloxone from the embedded MSN‐A/Nal upon fentanyl triggering. Overall, iNal shows suitable morphology, strength, skin tolerance, and swelling for responsive delivery.

#### 2.1.3 In Vitro Naloxone Release From the iNal Patch

Next, we evaluated the release characteristics of the fully assembled iNal patch incorporating the MSN‐A/Nal nanoparticles. Similar to the free MSNs, the iNal patch demonstrated fentanyl‐triggered naloxone release that was unaffected by the presence of FBS/BSA, further confirming its suitability for transdermal application (Figure 3J). The release kinetics from the iNal patch mirrored those observed with MSNs in solution, showing a burst release within the first 30 min. Notably, the cumulative amount of naloxone released from the iNal patch generally exceeded that from the equivalent amount of free MSNs under the same conditions. Specifically, at 10 µg/mL fentanyl, the iNal patch released 10.57 ± 1.11 µg/mL naloxone, increasing substantially to 128.26 ± 7.38 µg/mL at 100 µg/mL fentanyl, again demonstrating robust dose dependency (Figure 3K). The iNal patch also exhibited cyclical release upon repeated fentanyl exposure. Compared to the free MSNs, the cyclic release from the MN system appeared more consistent and sustained across cycles, with an approximate ∼10% reduction by the third cycle. This enhanced performance may be attributed to the potentially higher local concentration and controlled microenvironment for the MSNs within the swelling PVAMA matrix, ensuring a more stable and prolonged naloxone release over multiple triggering events (Figure 3L).

#### 2.1.4 In Vitro Evaluation of Naloxone Activity Using an Engineered Cell‐Based Sensor

**FIGURE 4:**
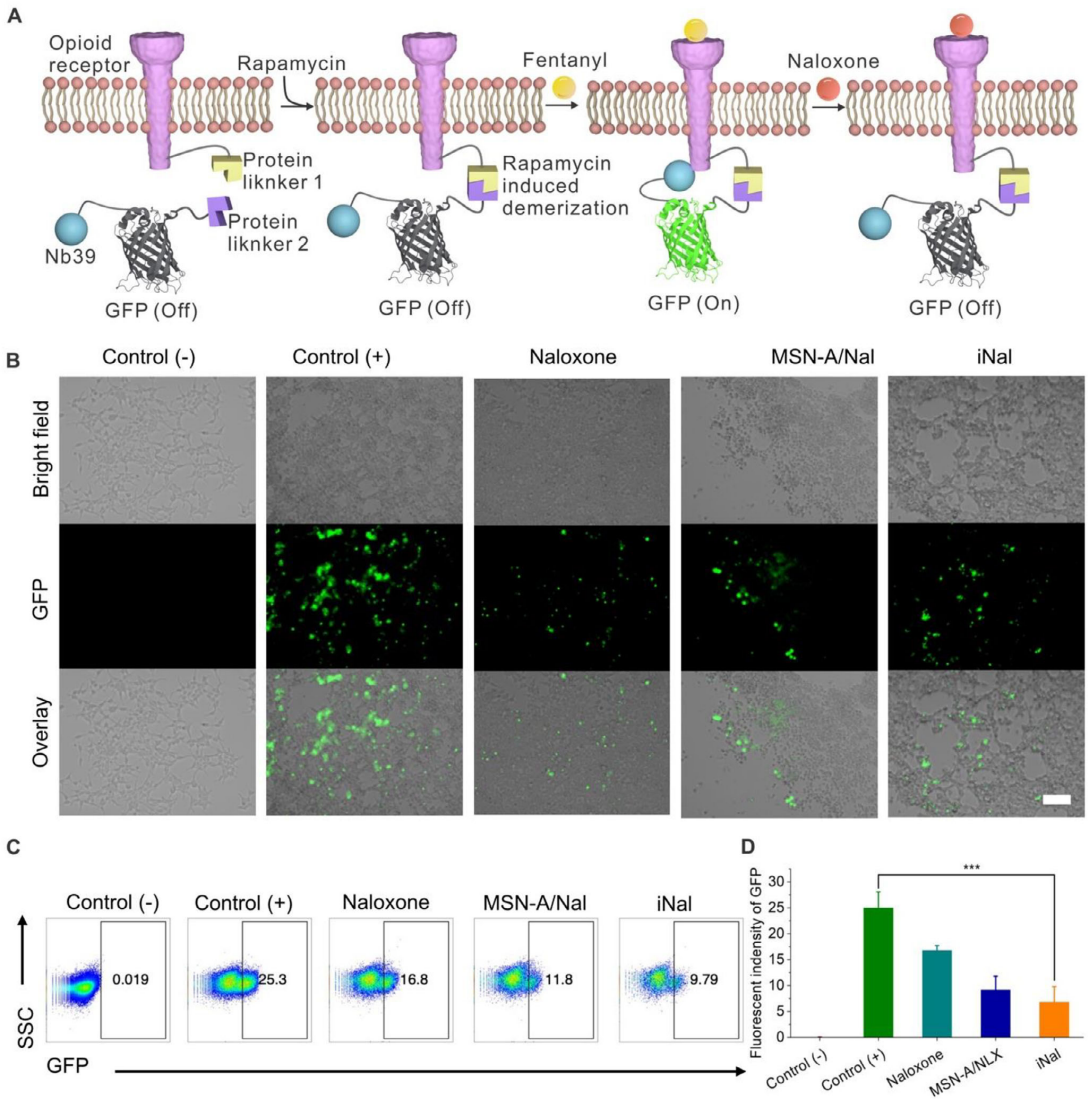
In vitro evaluation of MSNs and iNal. (A) Schematic of an engineered cell and treated by rapamycin, fentanyl, and naloxone. Nb39 means nanobody 39 [56]. (B) Confocal fluorescent images of HEK 293T cells when treated by different drugs. Scale bar, 100 µm. (C) Quantification of µ‐opioid receptor activation (percentage of GFP‐positive cells) in HEK293T cells after treated by different drugs, control (‐) means no transfection, control (+) means transfection and treated by rapamycin and fentanyl. Respective activation efficiencies are indicated in each square box. (D) Relative quantification of GFP in different groups. Data are presented as mean ± s.d. (n = 3). ***p < 0.001.

To validate the functional efficacy of the fentanyl‐triggered naloxone release from our MSN‐A/Nal and iNal patch platforms at a cellular level, we utilized an engineered HEK293T cell line expressing the µ‐opioid receptor alongside a rapamycin‐inducible green fluorescent protein (GFP) reporter system [35, 36]. As previously described, this system incorporates membrane‐anchored protein linkers designed to dimerize upon rapamycin treatment; subsequent activation of the µ‐opioid receptor by an agonist like fentanyl triggers conformational changes that facilitate GFP fluorescence reconstitution (Figure 4A). Conversely, naloxone, acting as a competitive antagonist with higher affinity for the µ‐opioid receptor, prevents fentanyl binding or displaces bound fentanyl, thereby inhibiting GFP signal generation. This engineered cell line thus serves as a dynamic biosensor to assess the effective concentration of naloxone released from our delivery systems in response to fentanyl [35, 36].

We first qualitatively assessed the system's response using confocal microscopy. As expected, cells treated with rapamycin and fentanyl exhibited clear GFP fluorescence, indicating µ‐opioid receptor activation (Figure 4B). In contrast, when cells were co‐treated with fentanyl and either free naloxone solution, fentanyl‐exposed MSN‐A/Nal nanoparticles, or fentanyl‐exposed iNal patch, the GFP signal was markedly reduced (Figure 4B). This visual evidence confirms that naloxone released from both the MSN‐A/Nal and iNal patch effectively antagonizes fentanyl activity at the µ‐opioid receptor, similar to the free naloxone control. For quantitative assessment, we employed flow cytometry to measure the percentage of GFP‐positive cells under different treatment conditions following rapamycin and fentanyl stimulation (Figure 4C). The positive control group (fentanyl only) showed a GFP fluorescence signal in 25.0% ± 3.1% of the cells. Treatment with free naloxone solution significantly reduced the GFP signal to 16.8% ± 0.9%. Notably, treatment with fentanyl‐stimulated MSN‐A/Nal or the iNal patch resulted in even stronger inhibition of the GFP signal, reducing the positive populations to 9.2% ± 2.6% and 6.8% ± 3.0%, respectively. This performance recapitulates the performance of the reported opioid biosensor.

To evaluate the biosafety of our MSNs and iNal patch, we tested human dermal fibroblasts (HDFs), human liver cancer cells (HepG2), mouse embryonic fibroblast cells (NIH 3T3), and transplantable rat pheochromocytoma (PC‐12) cells, related to the major potentially affected organs. No harm was observed in all the cells tested (Figure S7), suggesting the iNal patch exhibits good biocompatibility with skin tissues and major organs. Both MSN‐A/Nal and the iNal patch released active naloxone that was more effective at antagonizing fentanyl than free naloxone in this cellular assay. This enhanced effect might be attributed to the sustained or prolonged release profile of naloxone from the nanoparticle/MN systems, potentially counteracting naloxone degradation by cellular processes over the experimental duration, in contrast to a bolus dose of free naloxone.

#### 2.1.5 In Vivo Naloxone Release From the iNal Patch

**FIGURE 5:**
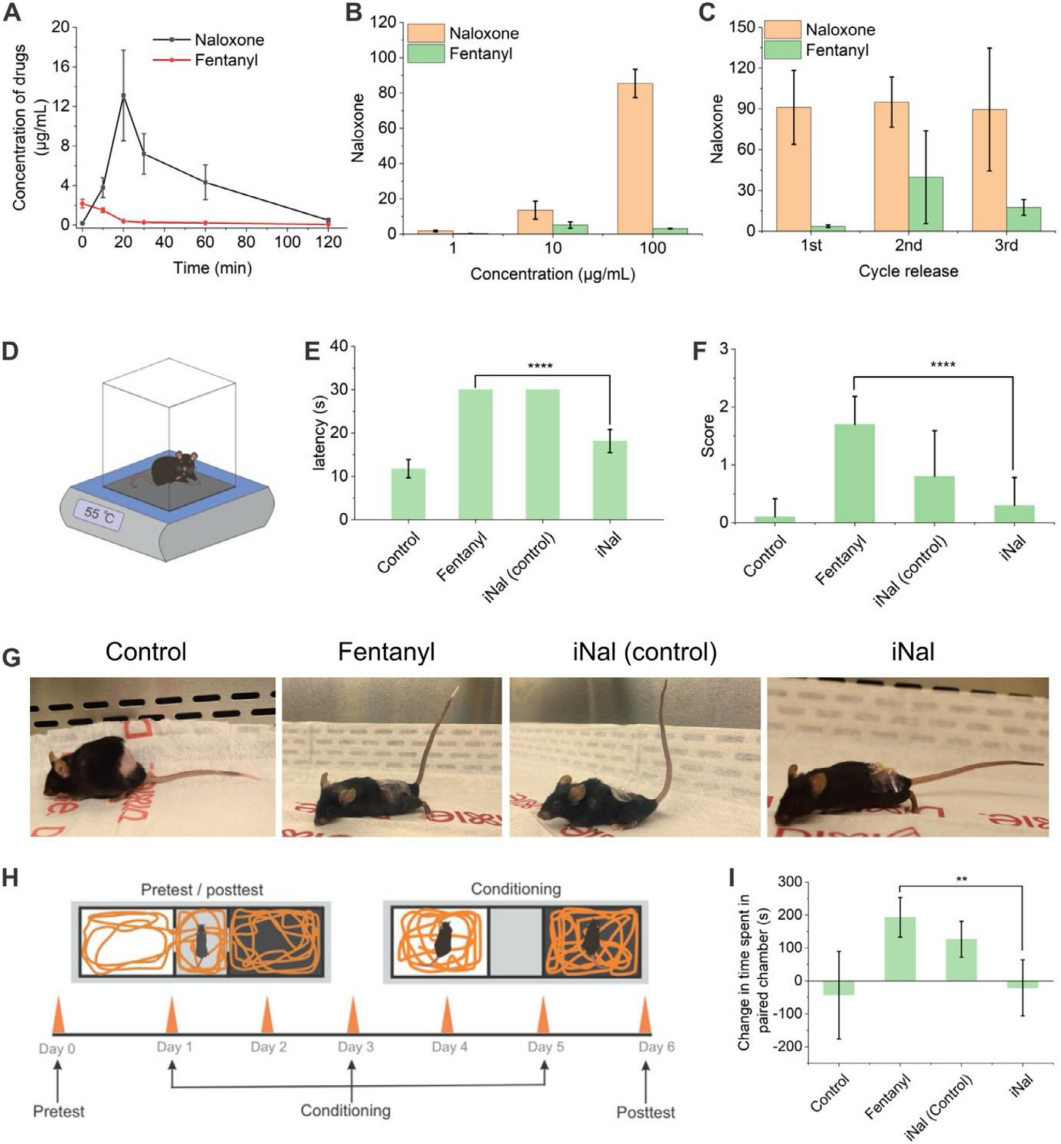
In vivo evaluation of iNal. (A–C) In vivo naloxone release profiles from microneedles (MNs). (A) Cumulative naloxone release over time following MN administration. (B) Naloxone release triggered by varying concentrations of fentanyl from the iNal patch. (C) Cyclic release behavior of naloxone in response to repeated fentanyl stimulation, demonstrating responsiveness and reversibility. Data are presented as mean ± s.d. (n = 3). (D,E) Evaluation of analgesic response using the hot plate test. (D) Schematic illustration of the experimental setup. (E) Thermal nociceptive thresholds of mice measured at 55°C, recorded as latency to nocifensive behaviors (hind paw licking or jumping), indicating the analgesic effect. Data are presented as mean ± s.d. (n = 10). (F,G) Assessment of fentanyl‐induced Straub tail response and its reversal by naloxone. (F) Quantitative scoring of Straub tail severity under different treatments. Data are presented as the mean ± s.d. (n = 10). (G) Representative images showing the Straub tail phenotype across different treatment groups. (H and I) Behavioral evaluation using the conditioned place preference (CPP) test. (H) Schematic of the CPP experimental design. (I) CPP results indicating drug‐associated preference under different treatment conditions, *p* = 0.004. Data are presented as the mean ± s.d. (n = 5). Statistical significance was determined by two‐tailed Student's t‐test (*p < 0.05, **p < 0.01, ***p < 0.001, ****p < 0.0001).

To confirm the therapeutic potential in vivo, we applied the iNal patch to mice and subsequently administered fentanyl systemically, monitoring serum naloxone and fentanyl levels over time using Liquid Chromatography‐Mass Spectrometry (LC‐MS). Following a high‐dose fentanyl injection (50 µg/kg) intended to model severe acute overdose, detectable serum naloxone levels emerged around 20 min post‐injection, reaching a peak concentration of 13.11 ± 4.59 µg/mL before gradually declining, likely due to drug metabolism and clearance paralleling the decrease in fentanyl levels (Figure 5A). This robust release profile confirms the patch's capacity to generate high systemic antidotal levels necessary to compete with high‐affinity opioids in acute bolus scenarios. This aligns with the critical need for autonomous intervention in unwitnessed overdose scenarios, where immediate antagonism is required before emergency responders can arrive. Consistent with in vitro findings, the in vivo naloxone release was also dose‐dependent; increasing the fentanyl challenge dose to 100 µg/mL resulted in a higher peak serum naloxone concentration of 85.44 ± 7.99 µg/mL (Figure 5B). Crucially, the iNal patch demonstrated sustained efficacy in vivo, enabling triggered naloxone release over at least three distinct fentanyl challenge cycles (Figure 5C). This is particularly important for real‐world harm reduction applications, acting as a prophylactic safety net for high‐risk individuals.

#### 2.1.6 In Vivo Behavioral Evaluation for Opioid Overdose in Mice

Encouraged by the promising in vitro findings, we next evaluated the therapeutic efficacy of the fentanyl‐responsive iNal patch in vivo, using established behavioral assays to measure the physiological and neurological effects of opioid overdose in mouse models. These experiments aimed to determine whether the controlled, responsive naloxone delivery via our iNal patch could effectively reverse opioid‐induced symptoms, compared to control conditions.

We first conducted the hotplate analgesia assay to quantitatively assess opioid‐induced analgesic effects and naloxone‐mediated reversal. Opioids like fentanyl significantly elevate thermal pain thresholds, which provides a robust indicator of opioid receptor activity. Mice were tested on a hotplate at a constant temperature of 55°C, and the analgesic effect of fentanyl was determined by measuring increased latency to nocifensive behaviors (hind paw licking or jumping). As illustrated in Figure 5D,E, fentanyl‐treated mice demonstrated significantly prolonged latency responses (exceeding 30 s), indicative of the analgesic effect of fentanyl. Post‐hoc analysis confirmed a statistically significant difference compared to the control group (*p* < 0.0001). Remarkably, treatment with the iNal patch substantially reduced this latency (18.2 ± 2.7 s) compared with fentanyl‐treated mice, demonstrating effective antagonism of fentanyl‐induced analgesia. In sharp contrast, control iNal patch (without functionalized nanoparticles) failed to reverse fentanyl‐induced analgesia, confirming that the specific nanoparticle‐mediated naloxone release was essential for reversing overdose symptoms. These findings not only underscore the pharmacological validity of the iNal patch but also confirm that the aptamer‐gated mechanism retains sufficient sensitivity to operate within the complex physiological environment of a living subject, overcoming the dilution effects inherent to systemic circulation. These findings are consistent with previous naloxone efficacy studies in opioid overdose models [37, 38], underscoring the pharmacological validity of the iNal patch.

As a secondary in vivo output measure, we evaluated the opioid‐induced Straub tail effects. Straub tail, a rigid upward‐curved tail, is a characteristic physiological response observed in opioid‐treated animals, correlating with opioid receptor‐mediated central nervous system activity [39]. Mice administered with fentanyl displayed pronounced Straub tail responses, post‐hoc analysis revealed a statistically significant difference compared to the control group (*p* < 0.0001), supporting the characteristic effect of fentanyl with average tail elevation angles of approximately 90° (Figure 5F,G). However, mice treated with the iNal patch exhibited a complete absence of the Straub tail response, maintaining normal tail posture comparable to untreated controls. Quantitative analysis further revealed significant differences between fentanyl‐alone and iNal patch‐treated groups (*p* < 0.001), clearly demonstrating naloxone's potent ability to antagonize fentanyl‐induced physiological changes via the responsive MN platform. These results strongly support the efficacy and specificity of the iNal patch in reversing fentanyl's CNS effects.

To further evaluate the impact of the iNal patch system on opioid‐induced behavioral preferences, we conducted a conditioned place preference (CPP) test [40, 41, 42, 43], a well‐established behavioral paradigm for measuring the rewarding and reinforcing properties of opioids (Figure 5H,I). In this assay, mice conditioned with fentanyl exhibited marked preferences for fentanyl‐associated chambers, spending significantly more time in these areas, reflecting strong opioid‐induced reinforcement. Post‐hoc analysis showed a statistically significant increase in time spent in drug‐paired chambers compared to controls (*p* < 0.01). Importantly, mice receiving the iNal patch did not display this preference, demonstrating substantially reduced exploration time in fentanyl‐conditioned chambers, nearly matching control levels. The significant attenuation of fentanyl‐induced preference behavior indicates successful naloxone‐mediated reversal of opioid reinforcement, further highlighting the potential of the iNal patch in mitigating the acute rewarding effects of fentanyl, a key component of comprehensive harm reduction. These outcomes align with prior reports of naloxone's effectiveness in reversing opioid‐conditioned behaviors [42, 44], validating the translational potential of our responsive MN platform.

Finally, biosafety remains a paramount concern for implementing transdermal drug delivery platforms in clinical applications. To address this, we performed comprehensive histological evaluations (H&E staining) of vital organs from mice treated with an iNal patch. No observable pathological changes or inflammatory responses were detected across various organs, including liver, kidney, heart, lung, and spleen, after MN administration (Figure S8). These histological results confirmed the high biocompatibility and systemic safety of the iNal patch, consistent with previously established safety profiles for similar transdermal MN systems [45, 46].

## 3 Conclusion

In this study, we developed an innovative, fentanyl‐responsive iNal patch designed for on‐demand naloxone delivery. This platform integrates two key technological advancements. First, we engineered MSNs loaded with naloxone and capped by fentanyl‐specific aptamers. The aptamer gates enable direct fentanyl sensing; fentanyl binding causes aptamer detachment and triggers proportional naloxone release [47, 48]. The mesoporous structure facilitates high drug loading (DLE 85.3%) and enables cyclic release by maintaining a high density of gated pores. This multi‐cycle capability is likely achieved through the sequential activation of the extensive reservoir of capped pores. Each fentanyl challenge unlocks only a fraction of the available nanopores, the remaining population of capped pores remains primed to respond to subsequent overdose events, a crucial feature for counteracting prolonged opioid effects or repeated exposures. Second, to enable convenient and efficient transdermal administration, these functionalized nanoparticles were incorporated into an MN array fabricated from PVAMA, a biocompatible and swellable polymer. The MNs exhibited robust mechanical integrity (failure force > 2.6 N), which facilitated effective skin penetration (∼400 µm depth) without needle fracture, reaching the interstitial fluid. Upon insertion, the PVAMA matrix rapidly swells (swelling ratio ∼200%), enhancing fluid uptake and facilitating the diffusion of fentanyl to the embedded nanoparticles and the subsequent release of naloxone into the tissue. This MN design overcomes the reliance on bystander intervention inherent to traditional injection or nasal spray methods and enables direct interaction with the physiological milieu for autonomous fentanyl sensing.

In vitro, the iNal patch showed robust, fentanyl concentration‐dependent naloxone release, sustained over 24 h with successful multi‐cycle triggering. It should be noted that the fentanyl trigger concentrations used in these characterization studies (up to 100 µg/mL) exceed typical clinical plasma levels. These elevated concentrations were employed to validate the material's responsiveness under saturation conditions and to demonstrate the high carrying capacity of the MN array, ensuring a sufficient safety margin for effectively reversing lethal doses in diverse overdose scenarios. However, the definitive validation of the system's sensitivity at physiologically relevant concentrations is provided by our in vivo studies. The systemic fentanyl challenge administered to mice results in serum concentrations orders of magnitude lower than the in vitro saturation tests, yet successfully triggered the aptamer‐gated mechanism. This confirms that the high‐affinity aptamers maintain their gating function and responsiveness even at the nanomolar concentrations characteristic of clinical overdose scenarios [49, 50]. Functional validation using an engineered cell‐based µ‐opioid receptor sensor confirmed that naloxone released from both MSN‐A/Nal and the iNal patch effectively antagonized fentanyl activity, potentially even more effectively than free naloxone, possibly due to sustained release profiles. Crucially, in vivo studies in mice confirmed the translational potential. Application of the iNal patch followed by systemic fentanyl challenge resulted in rapid, dose‐dependent appearance of naloxone in serum, demonstrating successful transdermal delivery and responsive release. The patch maintained its responsiveness over multiple fentanyl challenges in vivo. Furthermore, the iNal patch effectively reversed fentanyl‐induced behavioral symptoms in mice, including thermal analgesia, the Straub tail response, and conditioned place preference, validating its therapeutic efficacy. Comprehensive histological analysis indicated good biocompatibility with no observable pathology in vital organs. While these results are highly promising, further development is warranted. Future work could focus on validating the device against a broader panel of interfering opioids to confirm the chemical selectivity derived from the aptamer, and fine‐tuning the release kinetics by modulating the MSN structure or the MN polymer composition for specific clinical scenarios. Nevertheless, this work presents a significant advancement by demonstrating a device capable of direct opioid sensing coupled with autonomous, triggered antidote delivery. This platform offers a promising new strategy for autonomous harm reduction and holds potential for broader applications in stimuli‐responsive transdermal therapeutic systems. Despite the promising performance of the iNal system demonstrated in this study, several limitations should be acknowledged. First, transdermal drug delivery efficiency may be influenced by inter‐individual variability in skin permeability, including differences in skin thickness, hydration, and physiological condition, which could affect the consistency of naloxone release [51, 52]. In addition, although the aptamer‐based sensing mechanism enables fentanyl‐responsive release, the long‐term stability of the aptamer under physiological conditions and during storage remains to be systematically evaluated [53]. Potential degradation or loss of binding affinity over time may impact system reliability [54, 55]. Furthermore, practical considerations for real‐world deployment, such as environmental variability, user handling, and compliance, may also influence overall performance. Addressing these factors in future studies will be important to further optimize the robustness and translational potential of the iNal platform.

## Conflicts of Interest

W.S. and P.Z. have applied for a patent related to this study, and the other authors declare no conflicts of interest.

## Funding

This work was funded by VT BSE startup package.

## Supporting information




**Supporting File**: advs76154‐sup‐0001‐SuppMat.docx.

## Data Availability

All data needed to evaluate the conclusions in the paper are present in the paper and/or the Supplementary Materials.
