# Supplementary material for: A Fentanyl‐Responsive Microneedle Patch for Harm Reduction
Source: Adv Sci (Weinh). 2026 Jul 9;13(47):e24301. doi: 10.1002/advs.202524301 (PMC13348650; doi:10.1002/advs.202524301)
Supplement: Supplementary file 1 [file ADVS-13-e24301-s001.docx]

A Fentanyl-Responsive Microneedle Patch for Harm Reduction

Penghui Zhao, Zerui Zhou, Tyler Wolter, Huanqing Niu, Yuanzhi Bian, Chixia Tian, Chun Xu, Chenming Zhang, Juhong Chen, Matthew Buczynski, Wujin Sun*

**EXPERIMENTAL METHODS**

**Materials.** All of the chemicals were purchased from Sigma–Aldrich unless otherwise specified and were used as received. Oligonucleotides were purchased from Integrated DNA Technologies. The water used in the experiments was obtained from a Milli-Q system.

The sequences of nucleic acids used in this work were as follows:

The cDNA: 5’ - GTCGTAAGTTCTGCC –NH_2_-3’.

The fentanyl aptamer: 5’-TGGCAGAACTTACGACGAGCGCGTGTGGCCGGCGTGAGGGAGGTGAGTCGTAAG -3’.

The control DNA: 5’-AGGCAGAACTTACGACAGCGAGCAGCAAACGTAAATTCTCGGCGTGCTTGGTGT-3’.

**Preparation of functionalized mesoporous silica nanoparticles (MSN).** We have used a modified sol-gel approach to make MSNs as reported.[1] Briefly, 1.4 mL silane triethoxysilylpropyl succinic anhydride (TESPSA) was dissolved in 1 mL deionized water containing 0.27 mL HCl (c = 0.05 M) and hydrolyzed overnight under stirring at room temperature to obtain hyd-TESPSA. Then, 1.60 g Cetyltrimethylammonium bromide (CTAB) was added to a mixture of 60 mL anhydrous ethanol and 200 mL double-distilled water, and the system was stirred until clear at room temperature. 1.6 mL ammonia and 0.45 g hyd-TESPSA were sequentially added to the above solution under stirring. After stirring evenly, 5 mL Tetraethoxysilane (TEOS) was pipetted into the homogeneous solution under vigorous stirring. A white precipitate was obtained after stirring for 6 h and resting for 24 h. Then, the product was separated by centrifugation, washed with water and ethanol for three times, and dried in a vacuum drying oven at 60 ℃. The template was removed by refluxing in a methanol solution of 0.1 mol/L HCl for 4 h at its boiling temperature. The process is repeated three times to remove the template, and then MSN-COOH was obtained.

cDNA modification: For cDNA-modified MSN, carboxylated MSN (3.0 mg) was incubated with phosphate buffered saline (PBS, 1.0 mL, 1.0 mM, pH = 7.4) containing 1-ethyl-3-(3-dimethylaminopropyl) carbodiimide (EDC, 10 mg⋅mL^–1^) and *N*-hydroxy succinimide (NHS, 10 mg⋅mL^–1^). After 30 min, cDNA (34 nmol) of fentanyl aptamer[2] was added, and the mixture was gently shaken overnight. The cDNA-functionalized MSN was washed with PBS (1.0 mM, pH = 7.4) for three times, removing uncombined DNA.

Naloxone loading: The cDNA-functionalized MSN (10.0 mg) was transferred to Phosphate Buffered Saline (PBS, 2.0 mL, 1.0 mM, pH = 7.4) and incubated with naloxone for 24 h. Afterward, fentanyl aptamer was added, and the vial was placed under gentle shaking overnight, resulting in the locking of the loaded cDNA-functionalized MSN. Following that, the locked and loaded MSNs was centrifuged (9000 rpm, 5 min), washed with PBS (1.0 × 10^–3^ M, pH = 7.4) for three times to remove the excess naloxone, and then dispersed in PBS (2 mL, 1.0 × 10^–3^ M, pH = 7.4) to get MSN-A/Nal. MSN-N-C was synthesized using the same method, with the fentanyl aptamer replaced by control DNA.

Drug loading efficiency and drug loading capacity was calculated by the following equations (1) and (2),

$$\begin{aligned} Drug loading efficiency \left( DLE \right) = \frac{W_{loaded drug}}{W_{Drug}}\times100\%\#\left( 1 \right) \end{aligned}$$

$$\begin{aligned} Drug loading capacity \left( DLC \right) = \frac{W_{loaded drug}}{W_{total}}\times100\%\#\left( 2 \right) \end{aligned}$$

Where *W_loaded drug_* is the loaded weight of the naloxone, *W_drug_* is the weight of naloxone in total, and *W_total_* is the weight of naloxone and MSNs.

Zeta potential, size, and polydispersity index (PDI) were measured using a Malvern nano Zetasizer after samples were diluted in Milli-Q water. SEM and TEM were used for morphology and EDS. Dry samples were imaged on copper grids. We used the Brunauer-Emmett-Teller (BET) study and the Barrett-Joyner-Halenda (BJH) study to calculate the surface area and pore volume of MSNs. UV-Vis spectrum was used for detecting the DNA in MSNs.

**Naloxone loading/release profile.** High-Performance Liquid Chromatography (HPLC, Shimadzu LC-20) was used for naloxone quantification as reported.[3] We used a Shimadzu 20A HPLC system for isocratic elution with a ZORBAX® Eclipse plus 250 × 4.6 mm, 5 μm C18 column (Agilent, CA, USA). The mobile phase comprised 420:580:1 v/v/v methanol, sodium 1-octanesulfonate solution (1.36 g sodium 1-octanesulfonate with 1.0 g sodium chloride in 580 mL water), and phosphoric acid with a flow rate of 1 mL/min and a wavelength of 214 nm. A calibration curve was obtained for 0.1–200 μg/mL naloxone. To maximize naloxone loading, the fentanyl aptamer was hybridized onto the MSN after the uncapped MSN-cDNA was loaded with naloxone. The naloxone was loaded into the MSN-cDNA by mixing 1 mg of MSN-cDNA with 1 mL of a naloxone hydrochloride solution (20 mg/mL) and sonicating the mixture for 2 min. The mixture was stirred for 24 h in dark under 25 °C. Nanoparticles were collected via centrifugation (13000 rpm, 20 min), and HPLC measurements of the supernatant were used to determine drug loading efficiency and drug loading capacity.

**Preparation of MSN-loaded Microneedle (MN).** MN patches were fabricated using a micromolding technique with maleated Poly(vinyl alcohols) (PVAMA) as the matrix material.[4] PVAMA was synthesized by reacting Poly(vinyl alcohols) (PVA, Mw ~31,000 - 50,000, 98% hydrolyzed) with maleic anhydride under controlled conditions to enhance swelling and mechanical properties. The PVAMA solution (20% w/v) was thoroughly mixed with MSN-A/Nal nanoparticles at predetermined concentrations (1 mg/mL or 10 mg/mL) to ensure homogeneous dispersion. The resulting nanoparticle-polymer mixture was cast into polydimethylsiloxane (PDMS) MN molds containing a 11 × 11 array of conical cavities (600 µm height, 300 µm base diameter, 300 µm inter-needle spacing). The molds were centrifuged at 3,000 rpm for 5 min to ensure complete filling of the cavities and to eliminate air bubbles. The filled molds were dried at room temperature under vacuum for 24 h to promote solvent evaporation and physical film consolidation. After drying, the solidified MN arrays were carefully demolded and stored in a desiccator until further use. Morphological evaluation of the MNs was conducted using SEM to confirm needle integrity and MSN distribution within the matrix.

**MN mechanical strength test.** The mechanical strength of the MN patches was evaluated by measuring the force required to cause needle fracture upon compression against a solid surface.[5] Using a mechanical tester (Instron 5944) equipped with a 10.00 N load cell, the MN patch was pressed against a flat stainless-steel plate. The initial distance between the MN tips and the plate was set to 2.00 mm. The plate moved towards the MNs at a constant speed of 0.1 mm/min. The failure force was recorded as the force at which the MNs began to buckle under compression.

**MN swelling property assessment.** The swelling behavior of the PVAMA MN patches was determined by measuring their weight change upon hydration [6]. Initially, the dry weight (W_D_) of an MN patch was recorded. The patch was then immersed in 25 mL of distilled water at room temperature for 10 min. After immersion, the swollen patch was removed, excess surface water was carefully blotted off, and the swollen weight (W_W_) was measured. The swelling ratio was calculated as a percentage using the following formula (3):

$$\begin{aligned} Swelling Ratio = \frac{W_{W}-W_{D}}{W_{D}}\times100\%\#\left( 3 \right) \end{aligned}$$

Where *W_D_* is the initial dry weight of the MN and *W_W_* is the weight of swollen MN.

**In vitro evaluation of released naloxone activity via engineered reporter cells.** The biological activity of naloxone released from the MSN-A/Nal nanoparticles and iNal patch upon fentanyl triggering was assessed using an engineered HEK293T cell line.[7] This cell line co-expresses the human µ-opioid receptor and a rapamycin-inducible GFP reporter system, where µ-opioid receptor activation by an agonist (like fentanyl) in the presence of rapamycin leads to GFP fluorescence reconstitution. Naloxone competitively antagonizes fentanyl binding, thereby inhibiting the GFP signal. To evaluate the released naloxone, these engineered cells were stimulated with rapamycin and fentanyl. Test groups were co-treated with fentanyl and either free naloxone solution (as a control), the supernatant collected from MSN-A/Nal nanoparticles previously exposed to fentanyl, or the release medium collected from iNal patch previously exposed to fentanyl. The antagonistic effect of the released naloxone was evaluated qualitatively by observing the reduction in GFP fluorescence using confocal microscopy and quantitatively by measuring the percentage of GFP-positive cells using flow cytometry.

**In vitro cytotoxicity evaluation of MNs.** The biocompatibility of the iNal patch was evaluated in vitro using multiple human cell lines, including human dermal fibroblasts (HDFs), human liver cancer cell (HepG2), mouse embryonic fibroblast cell (NIH 3T3), and transplantable rat pheochromocytoma (PC-12). Cells were seeded in 96-well plates at a density of 1 × 10⁴ cells per well and cultured in appropriate growth media under standard conditions (37 °C, 5% CO₂). After 12 h of attachment, cells were treated with MN extracts prepared by incubating MN patches in culture medium (200 µL per patch) at 37 °C for 2 h. Cell viability was assessed using a MTT assay. Absorbance was measured at 570 nm using a microplate reader (BioTek Synergy HTX). Cell viability was expressed as a percentage relative to untreated controls. All experiments were performed in triplicate.

**In vivo pharmacokinetics of naloxone release.** The in vivo release kinetics and fentanyl-responsiveness of the iNal patch were evaluated in C57BL/6J mice under Virgina Tech Institutional Animal Care and Use Committee (VT IACUC)-approved protocols. Patches were applied transdermally to the mice, the patch was applied to the dorsal skin of mice (area ≈ 0.81 cm²). Prior to application, the hair at the site was shaved to ensure consistent skin contact. followed by systemic administration of fentanyl via intraperitoneal injection to trigger naloxone release. Blood samples were collected from the tail vein at specified intervals, centrifuged (1000 ×*g*) to obtain serum, and analyzed for both naloxone and fentanyl concentrations using LC-MS. Three types of pharmacokinetic studies were conducted: (1) Time-dependent release: Serum was collected at 0, 10, 20, 30, 60, and 120 min following a single fentanyl injection (50 µg/kg) to determine the release profile over time (n = 5 mice per timepoint). (2) Dose-dependent release: The effect of varying fentanyl challenge doses (5 µg/kg, 50 µg/kg, 500 µg/kg) on naloxone release was assessed by collecting serum 20 min post-fentanyl injection. (3) Cyclic release: The patch's ability to respond to multiple challenges was tested by administering three sequential fentanyl injections (50 µg/kg each) and collecting serum 20 min after each injection.

**Hotplate test.** The hotplate test was used to evaluate the analgesic effects of fentanyl and the reversal efficacy of naloxone released from the iNal patch. Male C57BL/6J mice (8 - 10 weeks old) were individually placed on a hotplate apparatus (Ugo Basile, Italy) maintained at a constant temperature of 55 °C. The latency to exhibit nocifensive behaviors-either hind paw licking or jumping-was recorded as the response time. A cutoff time of 60 s was imposed to prevent tissue damage.

Mice were randomly divided into treatment groups (n = 5 per group): control (saline only), fentanyl-treated (50 μg/kg), fentanyl + free naloxone, fentanyl + iNal patch, and fentanyl + non-functional iNal control. For iNal patch treatment groups, the patch was applied 5 min before fentanyl administration. Latency measurements were recorded at baseline and 15 min post-treatment. A shortened response latency following iNal patch application was interpreted as effective reversal of fentanyl-induced analgesia. All experimenters were blinded to the treatment groups during scoring to minimize potential bias.

**Straub tail measurement.** The Straub tail response, a characteristic physiological sign of opioid activity involving tail rigidity and elevation, was assessed to evaluate the neurological effects of fentanyl and their reversal. Following treatment administration, mice (n = 10 per group) were observed, and the tail posture was scored using a scale from 0 to 2 based on the angle of elevation from the horizontal plane and rigidity. A score of 0 indicated a relaxed tail with no elevation; a score of 1 indicated a rigid tail elevated between 1° and 10°; a score of 1.5 indicated rigidity at the base of the tail with an elevation between 11° and 45°; and a score of 2 indicated rigidity at the base with an elevation between 46° and 90°. Scores were recorded at specified time points to assess the effect of the treatments. All experimenters were blinded to the treatment groups during scoring to minimize potential bias.

**Conditioned place preference (CPP) assay.** The effect of the iNal patch on fentanyl-induced reinforcement was evaluated using a CPP protocol under an approved VT IACUC protocol. A three-chamber CPP apparatus (ENV-3013, Med Associates Inc.) was used. The experiment comprised three phases as previously reported.[8] Pre-testing (baseline preference): mice were initially allowed to explore all three chambers freely for 15 min following a 5 min adaptation period in the central chamber to determine baseline preference for either side chamber. Conditioning: over subsequent days, mice underwent 30 min conditioning sessions confined to one of the side chambers immediately following treatment administration. Post-testing (preference test): After the conditioning phase, mice were again allowed to freely explore all three chambers for 15 - 20 min (following a 5 min adaptation) to assess changes in preference for the drug-paired chamber. Four experimental groups (n = 5 mice/group) were tested. Negative Control: received saline injections. Positive Control: received fentanyl injections (200 µg/kg). iNal patch Treatment Group: wore the iNal patch and received fentanyl injections. Control patch group: wore a control (non-functional) iNal patch and received fentanyl injections. The time spent in each chamber during the pre- and post-testing phases was recorded and analyzed to determine the preference score. All experimenters were blinded to the treatment groups during scoring to minimize potential bias.

**Histological analysis.** To evaluate tissue responses and systemic biocompatibility of the iNal patch, histological examinations were performed using H&E staining. For local skin assessment, MN patches were applied to the dorsal skin of C57BL/6J mice for 10 min. Skin tissues were harvested immediately after patch removal and at subsequent time points (1 and 3 days post-application) to assess wound recovery and local inflammation. To assess systemic toxicity, major organs including liver, kidney, heart, spleen, and lung were collected from mice 72 h after iNal patch application. All tissues were fixed in 4% paraformaldehyde at 4 °C for 24 h, dehydrated, and embedded in paraffin. Sections (10 μm thick) were cut using a microtome, mounted on glass slides, and stained with standard H&E protocols. Stained sections were imaged using a brightfield microscope (Leica), and histopathological evaluation was conducted to identify signs of inflammation, necrosis, or other pathological alterations.

**Statistical analysis.** All quantitative data were processed and analyzed using Origin (OriginLab) and SPSS (IBM) software. Data are presented as mean ± standard deviation (s.d.) or mean ± standard error of the mean (SEM), as indicated in the figure legends. The number of replicates or animals per group (n) is specified in the corresponding methods sections or figure legends. Statistical significance for comparisons between multiple groups was determined by two-tailed Student’s t-test, although the specific test used may vary depending on the data distribution and comparison being made. A probability value (*p*-value) less than 0.05 was considered statistically significant. Significance levels are denoted in the figures as follows: **p* < 0.05, ***p* < 0.01, ****p* < 0.001, *****p* < 0.0001.

**Table S1.** Drug loading efficiency/capacity and texture properties of the samples before and after drug loading.

| **Samples** | **Size (nm)** | **PDI** | **Zeta Potential(mV)** | **S_BET_(m^2^/g)** | **V_P_(cm^3^/g)** | **D_P_(nm)** | **DLE** | **DLC** |
| --- | --- | --- | --- | --- | --- | --- | --- | --- |
| MSN-COOH | 261.2 ± 4.7 | 0.13±0.02 | -47.8 ± 2.1 | 1066 | 0.44 | 3.8 | - | - |
| MSN/Nal | 286.5 ± 5.8 | 0.27±0.01 | -55.2 ± 1.3 | 545 | 0.12 | 3.4 | - | - |
| MSN-A/Nal | 289.7 ± 3.2 | 0.19±0.01 | -65.1 ± 1.6 | 459 | 0.09 | 1.0 | 85.31 ± 0.97 | 42.65 ± 0.48 |
| MSN-C/Nal | 303.3 ± 5.5 | 0.33±0.02 | -78.2 ± 9.2 | - | - | - | 95.43 ± 2.01 | 47.72 ± 1.01 |

^*^ S_BET_ means surface area of BET, V_P_ means volume of porous, D_P_ means diameter of porous, DLE means drug loading efficiency, DLC means drug loading capacity.

**Figure S1.** (**A**) Particle size distribution of MSNs via dynamic light scattering. The insets show the aqueous solutions of MSNs. (**B**) SEM images of MSNs. Scale bar, 200 nm. (**C**) TEM images of MSNs. Scale bar, 50 nm.

**Figure S2.** (**A** to **C**) Pore size and pore volume distribution curves of (A) MSN-COOH, (B) MSN/Nal, and (C) MSN-A/Nal.

**Figure S3.** EDS mapping was used to examine the elemental distribution of MSNs, focusing on carbon (C), nitrogen (N), oxygen (O), silicon (Si), and phosphorus (P). Scale bar, 250 nm.

**Figure S4.** (**A** and **B**) Drug loading efficiency in different (A) ratios of aptamer and MSN (M/w) and (B) ratios of naloxone and MSN (w/w).

**Figure S5.** Mice skin after treatment with iNal patch.

**Figure S6.** H&E-stained sections of mice skin pre- and post-treatment with MNs. Scale bar, 100 µm.

**Figure S7.** Cell viability of various cell lines (HDF, HepG2, NIH-3T3, and PC-12) following incubation with MNs or MSN-A/Nal (n = 3). The "Control" group refers to cells cultured without exposure to MNs or MSN-A/Nal.

**Figure S8.** H&E-stained sections of mice skin pre- and post-treatment with MSN-A/Nal or iNal patches. Scale bar, 100 µm.

REFERENCES

[1]	a) C. Xu, L. Xiao, Y. Cao, Y. He, C. Lei, Y. Xiao, W. Sun, S. Ahadian, X. Zhou, A. Khademhosseini, Q. Ye, *Nano Research* **2020**, *13* (9), 2323, <https://doi.org/10.1007/s12274-020-2783-z>; b) Z. Zhao, P. Zhang, Y. Zhao, L. Wang, J. Zhang, F. Bu, W. Zhou, R. Zhao, X. Zhang, Z. Lv, Y. Liu, Y. Xia, W. Zhang, T. Zhao, D. Chao, W. Li, D. Zhao, *Nat. Protoc.* **2024**, <https://doi.org/10.1038/s41596-024-01073-0>; c) M. Lian, Z. Xue, X. Qiao, C. Liu, S. Zhang, X. Li, C. Huang, Q. Song, W. Yang, X. Chen, T. Wang, *Chem* **2019**, *5* (9), 2378, <https://doi.org/https://doi.org/10.1016/j.chempr.2019.05.023>; d) E. Bagheri, M. Alibolandi, K. Abnous, S. M. Taghdisi, M. Ramezani, *Journal of Materials Chemistry B* **2021**, *9* (5), 1351, <https://doi.org/10.1039/D0TB01960G>.

[2]	J. Canoura, Y. Liu, J. Perry, C. Willis, Y. Xiao, *ACS Sensors* **2023**, <https://doi.org/10.1021/acssensors.2c02463>.

[3]	A. Tijani, P. Dogra, M. J. Peláez, Z. Wang, V. Cristini, A. Puri, *Drug Delivery and Translational Research* **2023**, *13* (1), 320, <https://doi.org/10.1007/s13346-022-01202-w>.

[4]	W. Sukhlaaied, S.-A. Riyajan, *Journal of Polymers and the Environment* **2014**, *22* (3), 350, <https://doi.org/10.1007/s10924-014-0651-1>.

[5]	J. Yu, Y. Zhang, Y. Ye, R. DiSanto, W. Sun, D. Ranson, F. S. Ligler, J. B. Buse, Z. Gu, *Proc. Natl. Acad. Sci. U. S. A.* **2015**, *112* (27), 8260, <https://doi.org/doi:10.1073/pnas.1505405112>.

[6]	E. Laszlo, G. De Crescenzo, A. Nieto‐Argüello, X. Banquy, D. Brambilla, *Adv. Funct. Mater.* **2021**, *31* (46), 2106061, <https://doi.org/10.1002/adfm.202106061>.

[7]	a) K. E. Kroning, M. Li, J. Shen, H. Fiel, M. Nassar, W. Wang, *ACS Chem. Biol.* **2022**, *17* (8), 2212, <https://doi.org/10.1021/acschembio.2c00364>; b) K. E. Kroning, W. Wang, *Angew. Chem. Int. Ed.* **2021**, *60* (24), 13358, <https://doi.org/https://doi.org/10.1002/anie.202101262>.

[8]	a) K. Moussawi, M. M. Ortiz, S. C. Gantz, B. J. Tunstall, R. C. N. Marchette, A. Bonci, G. F. Koob, L. F. Vendruscolo, *Sci. Adv.* **2020**, *6* (32), eabc0413, <https://doi.org/doi:10.1126/sciadv.abc0413>; b) C. D. Bryant, K. W. Roberts, C. S. Culbertson, A. Le, C. J. Evans, M. S. Fanselow, *Drug and Alcohol Dependence* **2009**, *103* (1), 74, <https://doi.org/https://doi.org/10.1016/j.drugalcdep.2009.03.016>.
